# Problematic Use of Social Networks during the First Lockdown: User Profiles and the Protective Effect of Resilience and Optimism

**DOI:** 10.3390/jcm11247431

**Published:** 2022-12-15

**Authors:** Covadonga González-Nuevo, Marcelino Cuesta, José Muñiz, Álvaro Postigo, Álvaro Menéndez-Aller, Daria J. Kuss

**Affiliations:** 1 Department of Psychology, University of Oviedo, Plaza Feijoo s/n, 33003 Oviedo, Spain; 2 Faculty of Psychology, University of Nebrija, Calle Santa Cruz de Marcenado, 27, 28015 Madrid, Spain; 3 International Gaming Research Unit and Cyberpsychology Research Group, Psychology Division, Nottingham Trent University, Nottingham NG1 4BU, UK

**Keywords:** social networks, problematic social media use, social comparison, addictive social media use, resilience

## Abstract

During the first lockdown, there was an increase in time spent using Social Networking Sites (SNS), which should be studied, as well as problematic SNS use. The present study has three objectives: to evaluate (i) the differences across gender and age and SNS type in increased SNS use, (ii) problematic SNS use during lockdowns, and (iii) the protective role of resilience and optimism on problematic SNS use. A total of 1003 participants (75.5% women) over 18 years old participated (*M* = 42.33; *SD* = 14.32 years). The use of SNS before and during lockdown, anxiety, depression, life satisfaction and problematic SNS use were evaluated. A repeated measures ANOVA and four regression analyses were calculated for the first objective regarding increased SNS use. Another linear regression analysis was calculated for the second objective regarding problematic SNS use. A correlational analysis has been performed to assess the protective roles of resilience and optimism. Differences in the increased use of SNS were found between the two time points and between the different types of SNS. Higher use of Instagram and YouTube was related to younger age. Being female was associated with higher Instagram use. Significant problematic use was found to be associated with younger age but was not dependent on gender. Higher levels of resilience and optimism were related to a lower level of problematic SNS use. SNS use during lockdown needs to be studied in order to understand factors that may protect against undesirable psychological consequences and support prevention programs.

## 1. Introduction

In January 2020, the World Health Organisation [1] announced an international health alert due to the pandemic caused by the Coronavirus 2019 (COVID-19). As a result, the Spanish government declared a state of alarm and the confinement of the population on 16 March 2020. This confinement consisted of complete isolation of the population in their homes, which they were only allowed to leave if they needed to shop for basic necessities (e.g., food or medicine). The complete lockdown lasted until 26 April when, for the first time, children under the age of 14 years were allowed to go out for an hour to go for a walk or play sports. In this context of loneliness and emotional distress [2,3,4], social habits were radically modified as a consequence of physical distancing. During this time, for many, online Social Networks (SNS) have become one of the limited ways of communication. The increase in SNS and internet use during lockdown has reached unprecedented peaks, which has been confirmed by several studies in different countries [5,6,7]. However, it is not known which individuals have increased their SNS use, which socio-demographic characteristics they have or which SNS have seen the greatest increase in use.

In the usual pre-pandemic context, overall SNS use has been found to be related to younger age and, in some studies, to being female [8,9]. However, the latter result regarding gender remains controversial as other studies found no relationship [10]. Therefore, it is of interest to assess whether the increase in SNS use during lockdown has also been higher in women and younger people or, on the contrary, has been uniform regarding gender and age. It is furthermore conceivable that the increase in SNS use was not the same across all platforms, as the reasons for their use are very different [11]. In the pre-pandemic context, the most used SNS platform was Instagram, followed by Facebook, YouTube and, finally, Twitter [12]. During the pandemic, one of the most widely used SNS platforms was Twitter, given its usefulness for sharing short text, where a multitude of conspiracy theories went viral [13]. The use of YouTube as a source of information through videos also stood out [14], as well as Facebook and Instagram as a form of social comparison and social support [15]. Who uses each type of social network more, depending on gender and age? Instagram use tends to be higher among younger people and among women in the non-pandemic context [12]. Twitter use is also characteristic of younger people, specifically those aged 16 to 24, but with no differences between the sexes [16]. Facebook use is more prevalent among those aged 25–40 years [12] and among women [17]. However, this trend changes depending on the study, as there are others that find no correlation between gender and age with using Facebook more time [18]. Finally, YouTube use is more prevalent among younger people and among males than females [19]. Therefore, in addition to understanding whether SNS use during the pandemic has been different across age groups and gender, it remains to be seen whether there has been a different increase in use depending on the type of SNS used. Understanding which users (age and gender) have increased their use and which SNS have increased in use will enable confirming which users increased their time spent the most when using specific platforms. As a result, it will be possible to clarify who were the most frequent users and which platforms were mostly used. This information is needed, among other reasons, because the reasons for use and the characteristics of each application are different [11] and, in addition, each social network has different mental health risks [20,21,22].

Once we understand the extent of SNS use increase during lockdown across gender and age, we can ask the same question about problematic SNS use and compare the profiles. In other words, are there any differences across age and gender in problematic social network use? Is the user profile (i.e., gender and age) that spends the most time using SNS the same as those with problematic SNS use? For example, women use SNS for longer periods of time and use them in a more problematic way [23,24,25]. Regarding age, in general, addictive internet behaviors are more prevalent in young people [26,27,28]. Problematic SNS use is generally defined as SNS use that generates negative consequences in a person’s life [23]. Two lines of research on problematic SNS use can be identified: one group of authors understands problematic SNS use as a purely addictive problem [29,30,31,32] based on addictive symptoms such as salience or tolerance proposed by Griffiths [33], and another group of authors understands problematic use more fully as a problem of excessive use along with other pathological features, such as a preference for online communication [34]. One of the most prominent pathological features of SNS use is negative comparative use, in which the user feels inferior to others [35]. This way of measuring SNS use has been proposed by González-Nuevo et al. [36].

Based on this understanding of problematic SNS use (including an addictive and a comparative component), age and gender differences in the pre-pandemic context should be looked for independently in each component. On the one hand, no differences between ages have been found in addictive SNS use [23,37], and no studies have been conducted that analyze differences by age in comparative SNS use. Concerning gender, both comparative [38] and addictive use have been found to be higher in women [23,37]. Accordingly, it would be of interest to understand the extent to which these gender and age differences are similar or different in the context of the pandemic. Identifying problematic SNS users’ socio-demographic characteristics will enable the focus of research and prevention campaigns on these at-risk individuals.

Next, taking into account the increase in overall and problematic SNS use in the context of the lockdown, the last question to be resolved is how this problematic use affected the population’s mental health and which protective variables have lessened this problematic SNS use. In the pre-pandemic context, numerous studies have been conducted linking anxiety, depression and life satisfaction to problematic SNS use, both regarding addiction [39,40] and comparisons [21,41,42]. During lockdowns, a relationship has also been found between addictive SNS use and psychological distress [43,44] with mediating effects, such as fear of COVID-19 [45,46] and fatalism [47]. It has also been proposed that COVID-19 stress is related to addictive SNS use [48]. However, to our knowledge, no research has analyzed the mental health consequences of increased addictive use of SNS during lockdowns.

Regarding comparative SNS use, a study by Masciantonio et al. [15] found relationships between lower psychological well-being and negative affect during lockdowns. However, this study used a two-item questionnaire to conduct the comparison and did not assess stress and anxiety and life satisfaction in relation to comparative use. Additionally, a study conducted by Yue et al. [49] indicated a relationship between the role of social comparison around the COVID-19 situation, including items such as “When I see others who are struggling with the coronavirus, I am happy that I am doing well” [49], and measured a higher level of stress. Therefore, it is necessary to expand the results regarding the consequences of comparative SNS use.

Finally, it is worth highlighting the psychological variables that can serve a protective function in preventing problematic SNS use: resilience and optimism. Resilience is a character trait that protects against the impact that stressful events can have on people’s mental health [50,51]. Specifically, as applied to the web, there is the concept of “digital resilience,” defined similarly to resilience as the ability to cope with negative experiences that happen online [52]. This concept of digital resilience became crucial during the lockdown in which the contents of SNS were especially full of distressing and depressing information in relation to COVID-19, and yet users spent a lot of time using them [53]. This act of continuing to surf the web despite sad content has been called “doom-scrolling” [54]. In addition, increased use of SNS would expose users to a higher risk of problematic SNS use [55]. It is, therefore, particularly relevant in this context to know whether resilience has been able to protect against problematic uses of SNS and COVID-related SNS use. In fact, resilience has already been shown to be a protective variable in the relationship between problematic SNS use and distress [56], between time spent and distress in the pandemic context [57] and as a way of dealing with lockdown [58].

On the other hand, optimism is defined as the tendency to think that the future can bring positive and favorable situations [59]. As with resilience, optimism has been related to a lower level of emotional distress [60] and has protected against burnout in situations of chronic stress [61]. Therefore, it makes sense that in a time of stress when SNS were used to a greater extent and depressing information took over SNS, people with high optimism would manage to make neither a problematic use of SNS nor a COVID-19-focused use of SNS that would take them away from a positive situation. Specifically, in relation to SNS use, optimism has also functioned as protective in the context of the relationship of comparative SNS use with psychological distress outside the pandemic context [62]. During the pandemic, the protective role of emotion regulation strategies in moderating the relationship between overall SNS use and COVID-focused SNS use was studied [63], as was the moderating role of mindfulness [46,64]. The moderating role of resilience in the relationship of overall SNS use with happiness level was also investigated [65], as well as the mediating role of positivity between problematic SNS use and anxiety level [66]. However, to our knowledge, the protective roles of resilience and optimism have not been included in studies of problematic SNS use during lockdowns in any country. Understanding the protective factors of adverse SNS use effects in a pandemic context, which is radically different from our non-pandemic lives, in which we were forced to use social networks to communicate, can help answer the question: Will resilience and optimism help to decrease the level of problematic SNS use and COVID-related use in a context of isolation? This may provide insight into how to protect oneself from inappropriate SNS use, even in a pandemic scenario.

Altogether, SNS use has considerably increased during lockdowns and generated a wealth of scientific research both with data collected directly from SNS and with questionnaires to users. This research has provided considerable evidence of the adverse mental health effects of problematic SNS use in isolation [15,43,44]. However, few studies assessed the use of SNS the way it has been assessed in the present study. Specifically, to the authors’ knowledge, no study has been conducted that exhaustively analyzed the increase in overall SNS use according to age and gender and the type of SNS used and compared this information with gender and age differences in the level of problematic use of SNS. Finally, no studies have assessed the protective role of resilience and optimism in preventing the problematic use of SNS.

Within this context, this research had the objective of understanding the characteristics of users who increased general and problematic SNS use during the first COVID-19 lockdown, as well as possible protective variables to prevent the consequences of problematic use in these users. This general objective is broken down into three specific objectives, to assess (i) differences depending on gender and age and the type of SNS in the increase of overall SNS use during the pandemic; (ii) whether there were gender and age differences in the level of problematic SNS use during lockdown; and (iii) the possible protective role of resilience and optimism on the level of problematic use of SNS.

## 2. Methods

### 2.1. Participants

The sample was initially composed of 1059 participants from the general Spanish population. The inclusion criteria for participation in the study were that participants had to be at least 18 years old and be SNS users. The final sample was reduced to 1003 persons after eliminating 5.29% of the sample for having more than two mistakes on the attentional control scale (described in the Instruments section). No missing data were obtained as all questions in the online questionnaire were mandatory. Participant ages ranged from 18 to 83 years (*M* = 42.33; *SD* = 14.32), and 75.5% of the sample were women (Table 1). In terms of educational level, 64.81% of the participants had a university education, 15.25% had Vocational Training, 13.46% had a Bachelor’s degree, 3.89% had completed Compulsory Secondary Education, and 2.59% had completed Primary Education.

### 2.2. Instruments

Problematic Use of SNS Questionnaire (PUS) [36]. This is a self-report consisting of 18 Likert-type items divided into 2 subscales on problematic SNS use. The first scale, Addictive Consequences (A.C.), consists of 10 items and assesses addictive SNS use. The second scale, Negative Social Comparison (N.C.), is composed of eight items and measures the degree to which the person, through the use of SNS, compares him/herself with others, believing him/herself to be inferior to them. All items have 5 response options, where 1 indicates “strongly disagree” and 5 “strongly agree.” In the present study, the internal consistency of the N.C. and A.C. scales was excellent (α = 0.94 and α = 0.91, respectively).

Brief Resilience Scale (BRS) [67]. This instrument assesses resilience, defined as the ability to recover from adversity and stress with a total of six items on a Likert scale with 5 scored response options, where 1 is “Strongly Disagree,” and 5 is “Strongly Agree.” The Spanish version has good internal consistency, with an alpha coefficient of 0.83 [68]. The α coefficient found in the present study was 0.85.

Optimism. It was assessed with the optimism subscale of the Entrepreneurial Personality Evaluation Battery (BEPE) [69]. It consists of 10 Likert-type items, with five response alternatives, where a higher score refers to greater optimism. The internal consistency of the subscale is 0.92 [69]. The α-coefficient found in the present study was 0.92.

Satisfaction with Life Scale (SWLS) [70]. This instrument is a life satisfaction scale consisting of five items. Participants are asked to indicate their level of agreement with each statement using a 5-point Likert scale (from 1 = strongly disagree to 5 = strongly agree). The estimated reliability using the α-coefficient in its Spanish adaptation is 0.88 [71]. The α-coefficient found in the present study was 0.82.

Hospital Anxiety and Depression Scale (HADS) [72]. The Spanish adaptation of Terol et al.’s HADS [73] was used. It is a 14-item questionnaire with 2 subscales of 7 items, each on a Likert scale ranging from 0 to 3. The subscale HADS-A assesses the level of anxiety. The other subscale, HADS-D, assesses the level of depression. A higher score means more severe anxiety and depression, respectively. The internal consistency for both scales in the Spanish version was 0.86 [74]. The α-coefficient found in the present study was 0.84 for the HADS-A subscale and 0.77 for the HADS-D subscale.

Overall use of SNS before lockdown. Time spent using the main SNS such as Facebook, Instagram, Twitter and YouTube before the lockdown was assessed through four Likert-type items worded as follows: “Before lockdown, how much time did you spend using Facebook on any given day?” and with six response options, “5 min or less”, “30 min”, “1 h”, “3 h”, “6 h” and “8 h or more”.

Overall use of SNS since lockdown. Time spent using the popular SNS YouTube, Facebook, Instagram and Twitter during confinement was assessed through four Likert-type items worded as follows: “Since lockdown, how much time did you spend using Facebook on any given day?” and with 6 response options, “5 min or less”, “30 min”, “1 h”, “3 h”, “6 h” and “8 h or more”.

Attentional control scale. A 10-question attentional control scale was included in which participants were asked to select a certain response option (e.g., In this question, you should select strongly agree). This scale was applied to detect those participants who responded randomly to the different questionnaires.

### 2.3. Procedure

A snowball sampling procedure was used to obtain the sample using different SNS (Facebook, Instagram, WhatsApp, and Telegram). The data were collected during the first lockdown of the Spanish population from 28 April to 7 May 2020. Data collection was done through an online questionnaire via Google Forms, anonymously and voluntarily, with participants giving their informed consent before starting. Both the items of the questionnaires and the attentional control scale were presented in a randomized order. Participants did not receive any reward for participating in the study.

### 2.4. Data Analysis

To assess the first objective, namely increased use of SNS, differences in the use of the most popular SNS (Facebook, Instagram, Twitter and YouTube) between pre-lockdown and during lockdown were analyzed using a repeated measures ANOVA. The two factors were (i) the four SNS and (ii) the time before lockdown and during the lockdown. The dependent variable was time spent using SNS. When statistically significant differences were observed as a function of the interaction between time spent on and type of SNS, the Bonferroni post hoc test was used to determine between which groups the differences occurred. To calculate the effect size, partial eta squared was used, with values from 0.010 to 0.039 considered a small effect size, from 0.040 to 0.110, moderate, and from 0.111 to 0.200 large [75].

In order to find out if this increase has been affected by gender and age, 4 linear regressions were performed predicting the increase in usage of each SNS in each (Facebook, Instagram, Twitter and YouTube). This score was calculated by subtracting the usage score of each SNS before the pandemic from the usage score during the pandemic, thus giving an incremental usage score for Facebook, Instagram, Twitter and YouTube. These direct scores were standardized for regression purposes. The coefficient of determination (R^2^) was used to determine the percentage of variance explained.

To find out the relative importance of gender and age in the problematic use of SNS during the pandemic as measured by the PUS (Objective 2), 2 linear regressions were performed with the A.C. subscale’s problematic use score and the N.C. score as dependent variables and gender and age as independent variables. The coefficient of determination (R^2^) was used to determine the percentage of variance explained.

To analyze the possible protective role of resilience and optimism on the level of problematic use of SNS as well as anxiety, depression and life dissatisfaction, correlation analyses were performed (Objective 3).

All analyses were performed with IBM SPSS v.24.

## 3. Results

### 3.1. First Objective: Age and Gender Differences in Increased SNS Use

We examined whether there were statistically significant differences in SNS use before and during the lockdown and between the type of SNS using a repeated measures ANOVA in which time spent using SNS was the dependent variable and the two time points the independent variable. The main effect of the type of SNS was statistically significant (*F*_(3,1000)_ = 165.14, *p* ≤ 0.001), with a large effect size (partial eta^2^ = 0.331). The main effect of time spent was statistically significant (*F*_(1,1002)_ = 774.23, *p* ≤ 0.001), with a large effect size (partial eta^2^ = 0.436), and the interaction was significant (*F*_(3,1002)_ = 68.96, *p* ≤ 0.001) with a large effect size (partial eta^2^ = 0.171). Table 2 below shows the significant differences in the increase in usage between the different SNS. There were significant differences between all SNS at time 1 (before confinement) except between Instagram and YouTube and also at time 2 (during confinement) except between Facebook and YouTube. At the pre-lockdown moment, Facebook was used significantly more than Instagram, Twitter and YouTube. On the other hand, Instagram was significantly more used than Twitter but not significantly different from YouTube. Finally, Twitter was significantly less used than Facebook, Instagram and YouTube.

**Figure 1:**
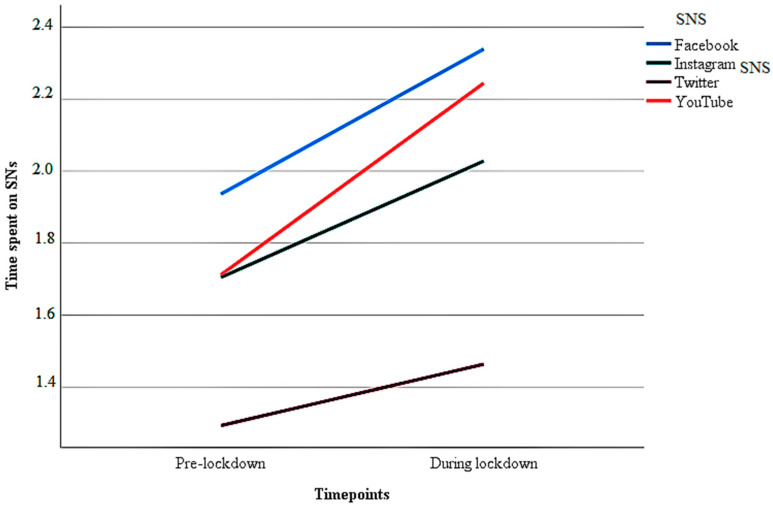
Graph of SNS Usage Time Depending on the Type of SNS Used at the Pre-Lockdown Time and During Lockdown.

Regarding the time point during the lockdown, Facebook was again the significantly more used SNS compared to Instagram and Twitter, but with no significant difference to YouTube. All SNS had a significant increase in time spent during lockdown compared to the pre-lockdown use. YouTube was the social network with the most marked increase in usage, followed by Facebook, Instagram and Twitter. In Figure 1, the differences can be seen more clearly in graphical form.

Since the first objective of this study was to reveal the potential predictive power of gender and age of increased use of Facebook, Instagram, Twitter and YouTube, four linear regression analyses were performed. The first regression equation to predict increased Facebook use was not significant (*F*_(2,1000)_ = 1.88, *p* = 0.154). The second regression equation to predict increased use of Instagram was significant (*F*_(2,1000)_ = 52.53, *p* < 0.001). The third regression equation to predict increased use of Twitter was not significant (*F*_(2,1001)_ = 0.988, *p* = 0.373), and the fourth regression equation to predict increased YouTube use was significant (*F*_(2,1001)_ = 13.26, *p* < 0.001). Regarding the results of increased Instagram use, the two predictors, age and gender, were able to explain 9.5% of the variance in increased Instagram use. Regarding the results of increased YouTube use, the predictor age was able to explain 2.6% of the variance. Table 3 shows that the two predictors contributed to the prediction of increased Instagram use, and only age contributed to predicting increased YouTube use. Age had negative values in both regression equations, indicating lower age predicted higher increased use of YouTube. Gender had a positive value, indicating that women had higher increased use of Instagram.

### 3.2. Second Objective: Age and Gender Differences in Problematic SNS Use

Since the second objective of this study was to reveal the potential predictive power of gender and age on problematic SNS use (A.C. and N. C.), two linear regression analyses were performed. The first regression equation to predict the level of A.C. was significant (*F*_(2,1000)_ = 37.29, *p* < 0.001), and the second one predicting the level of N.C. was also significant (*F*_(2,1000)_ = 19.93, *p* < 0.001). Only age was a significant predictor with negative values in both equations, explaining 6.9% of the variance in A.C. and 3.8% of the variance in N.C. (Table 4). This means that being younger predicted higher A.C. and N.C.

### 3.3. Objective Three: Protective Role of Resilience and Optimism on the Level of Problematic Use of SNS

A series of bivariate correlations between the different variables of the study were carried out, the values of which are presented in Table 5. As can be seen, there was a positive and significant correlation between the PUS subscales and the variables of depression and anxiety and a significant negative correlation between the PUS subscales and life satisfaction. Moreover, there was a significant and negative correlation between the PUS variables and resilience and optimism.

## 4. Discussion

This research had the objective of understanding the characteristics of users who increased general and problematic SNS use during the first COVID-19 lockdown, as well as possible protective variables to prevent the consequences of problematic use in these users. This general objective was broken down into three specific objectives, namely to assess (i) differences depending on gender and age in the increase of general SNS use during the pandemic and depending on the type of SNS (Facebook, Instagram, Twitter or YouTube); (ii) whether there were gender and age differences in the level of problematic use during lockdown; and (iii) the possible protective roles of resilience and optimism on the level of problematic SNS use.

Regarding the first objective, it has been found that there was an increase in overall SNS use during the lockdown with a large effect size, supported by other studies [5,6,7]. In addition, significant differences have been found between the types of SNS with a large effect size. Not only was there a significant increase in overall SNS usage time, but there were differences between the different types of SNS (Facebook, Instagram, Twitter and YouTube). In particular, there were significant differences between all SNS at time 1 (before lockdown), except between Instagram and YouTube, and also at Time 2 (during lockdown), except between Facebook and YouTube. Facebook was the most used SNS, followed by YouTube, Instagram and finally, Twitter at both Times 1 and 2.

There was a marked increase in YouTube use compared to the other SNS. YouTube, which was already widely used by the population as the second most used SNS [12], may have had a more marked increase as it is an entertainment SNS as well as informative [76]. Specifically, during lockdowns, this double aspect can be appreciated as, on the one hand, studies have been carried out on the informative capacity of YouTube on COVID [14,77] and, on the other hand, although to a lesser extent, YouTube use for recreational purposes (watching cooking videos, sports videos, etc.) [78,79]. On the contrary, this research found that there was a large percentage of people who maintained the same use of Twitter during lockdowns in comparison to pre-pandemic levels. Twitter focuses on generating information and discussion on different topics. The difference with YouTube is that Twitter is more focused on information and ideological discussion than on entertainment. Therefore, these results indicate that the population tended to use SNS aimed at sharing personal content (Facebook and Instagram) and entertainment content such as YouTube more than SNS aimed at discussion and information (i.e., Twitter). Although there was no significant increase in the population’s use of SNS, the amount of fake news and conspiracy theories on Twitter did increase [13].

Moreover, with regard to differences across age and gender in increased use of Facebook, Instagram, Twitter and YouTube, four linear regressions were calculated. Only the regression equation of Instagram and YouTube was significant. Only the variable age predicted increased YouTube use, which indicates that being younger predicted higher increased YouTube use. Moreover, both age and gender predicted increased Instagram use, indicating younger age and being female predicted higher increased Instagram use. Both results are consistent with previous studies, which indicated that being younger was related to higher SNS use [8,9]. Given that the SNS with the greatest increase was YouTube and that this increase was significantly greater among young people, it can be deduced that there was an increase in some SNS and among younger people. Therefore, in future studies, SNS should be studied, differentiating between SNS types since, as has been seen in this study, there are differences depending on the specific SNS.

With respect to gender, a different trend was observed depending on the type of SNS, with women being more likely to increase their Instagram use than men and younger people more likely to increase their Instagram use than older people. This result is in line with previous studies that found young women used SNS significantly more [23,25]. One possible explanation for why women use Instagram more than men is the motives behind their use. While women use SNS such as Instagram to maintain interpersonal relationships and to browse social information (i.e., information about other people, both intimate and acquaintances, such as news, posts and opinions), men use other SNS to browse general information such as news, entertainment and sport, among others [80]. However, to our knowledge, there are no studies that take into account gender differences depending on the specific type of SNS. This may be one of the reasons for the inconsistent results.

Regarding the second objective, the two regression equations of A.C. and N.C. were significant, and only age was a significant predictor. Specifically, the results indicated that being younger predicted higher A.C. and N.C. From these first two results, the sex variable was not relevant for predicting SNS use (except for Instagram use) or problematic use. This result conflicts with previous studies that found differences in favor of women [23,25]. Regarding age, it is striking that only for YouTube and Instagram did age have a significant weight, while age was a relevant variable for A.C. and N.C. With regards to A.C., the same trend has been found for problematic use and younger age in previous studies [28]. In the case of N.C., there are no studies that have verified this trend. This may mean that problematic SNS use is strongly related to a lower age, and nevertheless, time spent using SNS is related to a lower age depending on the type of social network. One possible explanation may be that problematic use is more frequent for one SNS (in this case, YouTube and Instagram) than another; however, to be able to conclude this, future research needs to study problematic SNS use differentiating between SNS. In this sense, existing evidence already supports this hypothesis. For example, the study by Pittman & Reich [81] found that the negative effect on psychological well-being resided in image-based SNS (i.e., Instagram) rather than in text-centered platforms (i.e., Twitter).

Regarding the third objective, a significant correlation was found between the different psychological variables studied and problematic use. A positive correlation was obtained with anxiety and depression, and a negative correlation with life satisfaction in A.C., a result in line with previous studies [39,40], and with N.C., a result also similar to previous studies [21,41]. Moreover, there was a negative correlation between the levels of resilience and optimism with both subscales, and these results are in line with previous studies in the pre-pandemic context [56,62]. Given this negative correlation, both resilience and optimism can be considered protective variables. To our knowledge, SNS use in relation to the protective roles of resilience and optimism has not been studied previously, although the negative relationship of N.C. with well-being has already been indicated by previous research [15].

The presented results must be interpreted considering some limitations. The comparison of usage time prior to and during lockdown is based on self-report in which participants were asked to recall their previous use, leading to recall bias risk. Data were collected during the lockdown and where individuals may have experienced atypical levels of stress and anxiety and SNS use. This creates the possibility of understanding human behavior with respect to SNS in situations of extreme stress in a unique way that could not have been studied previously. Moreover, the present sample consisted of people over 18 years, whilst research has found that adolescents under 18 years are more likely to be problematic SNS users [82]. Besides, most of the sample were highly-educated females, which limits the generalizability of the results to other populations, especially men. Future studies should use random sampling or, otherwise, at least undertake a representative sampling of the population by increasing the number of people with basic training and men. Given the cross-sectional study design, causal associations cannot be provided.

The presented findings have implications for the prevention of problematic SNS use in general and in contexts of complete isolation, which can be useful for multiple situations as well as generate valuable information on SNS use. Given that there was an increase in general and SNS problematic use during the lockdown, we need to understand how to prevent it from having negative psychological consequences on the population. This increase can recur in other situations of isolation for multiple reasons, not only during lockdowns, so these results can be extrapolated to other contexts. In these cases, it should be borne in mind that the increase in time spent using SNS is different depending on the type of SNS. Our findings indicate that the increase is higher for YouTube, followed by Facebook, which also is the most used social network in isolation and without isolation. Future studies should investigate why there is a preference for using these two SNS to communicate in isolation. It is also a relevant finding that users will differ depending on the social network, with a greater increase in young people using Instagram and YouTube as well as resilience and optimism may be protective. Therefore, if this or similar situations were to occur in the future, it would be advisable to carry out awareness-raising campaigns on how to make appropriate use of SNS in order to prevent problematic SNS use.

## 5. Conclusions

Altogether, there are significant differences in the time spent using SNS during the lockdown and previously. This increase was most striking for the SNS YouTube, followed by Facebook, Instagram and Twitter. It is also worth highlighting that Facebook was the most used SNS during the lockdown and previously. This increase in the case of Facebook and Twitter is not influenced by gender and age; however, in the case of YouTube use, the increase is greater the younger the age, and Instagram use increases more among young women. With respect to problematic use, both addictive and comparative use are predicted by a younger age regardless the gender. Finally, resilience and optimism play a protective role against problematic SNS use.

## Figures and Tables

**Table 1:** Sociodemographic Characteristics.

	Men		Women	
Age Ranges ^a^	*n*	% Total	*n*	% Total
18 to 24 years	34	3.4	89	8.9
25 to 40 years	69	6.9	279	27.8
41 to 55 years	83	8.3	257	25.6
55 or more	60	6.0	132	13.2
Total	246	24.5	757	75.7

Note. ^a^: The cut-off points were established using the intervals indicated in IAB Spain [12], as they found significant differences in the type of SN use used in each of the generations.

**Table 2:** Pairwise Comparisons Depending on the Type of SNS Used at Pre-Lockdown and During Lockdown.

	SNS Type (1)	SNS Type (2)	Mean Differences (1–2)	Sig.
Time 1: Pre-lockdown	Facebook	Instagram	0.231	≤0.001
	Facebook	Twitter	0.643	≤0.001
	Facebook	YouTube	0.224	≤0.001
	Instagram	Twitter	0.412	≤0.001
	Instagram	YouTube	−0.007	1.00
	Twitter	YouTube	−0.419	≤0.001
Time 2: During lockdown	Facebook	Instagram	0.311	≤0.001
	Facebook	Twitter	0.875	≤0.001
	Facebook	YouTube	0.095	0.287
	Instagram	Twitter	0.564	≤0.001
	Instagram	YouTube	−0.216	≤0.001
	Twitter	YouTube	−0.781	≤0.001

**Table 3:** Regression Equation Predicting the Increased Use of Facebook, Instagram, Twitter and YouTube.

Dependent Variable	Predictor	*B* (*SE*)	*β*	*T*	*Sig*
Increased Facebook use	Gender	0.08 (0.07)	0.04	1.13	0.258
	Age	−0.003 (0.002)	−0.05	−1.49	0.137
**Increased Instagram use**	**Gender**	**0.16 (0.07)**	**0.07**	**2.34**	**0.019**
	**Age**	**−0.02 (0.002)**	**−0.30**	**−9.79**	**<0.001**
Increased Twitter use	Gender	−0.09 (0.07)	−0.04	−1.17	−243
	Age	0.002 (0.002)	0.022	0.698	0.485
**Increased YouTube use**	Gender	0.035 (0.07)	0.015	0.481	0.630
	**Age**	**−0.011 (0.002)**	**−0.159**	**−5.08**	**<0.001**

Note. Significant results are highlighted in bold.

**Table 4:** Regression Equation Predicting the Level of Problematic SNS use.

Dependent Variable	Predictor	*B* (*SE*)	*β*	*T*	*Sig*
Addictive Consequences	Gender	−0.63 (0.44)	−0.04	−1.45	0.148
	**Age**	**−0.11 (0.01)**	**−0.26**	**−8.59**	**<0.001**
Negative Comparison	Gender	−0.31 (0.51)	−0.02	−0.61	0.543
	**Age**	**−0.10 (0.02)**	**−0.20**	**−6.31**	**<0.001**

Note. Significant results are highlighted in bold.

**Table 5:** Pearson Correlations Between the Social Comparison Subscale, the Addictive Behaviors Subscale, the COVID-Related SNS Use, the HAD Questionnaire, the SWLS Scale, the BRS Scale and the Optimist Subscale.

	Anxiety	Depression	Satisfaction	Resilience	Optimism
Addictive Consequences	0.31 **	0.28 **	−0.21 **	−0.23 **	−0.20 **
Negative Comparison	0.39 **	0.41 **	−0.37 **	−0.35 **	−0.36 **

** *p* ≤ 0.001.

## Data Availability

The datasets generated and analyzed during the current study are available from the corresponding author upon reasonable request.
